# Oxidative stress in the etiology of age-associated decline in glucose metabolism

**DOI:** 10.1186/2046-2395-1-7

**Published:** 2012-11-01

**Authors:** Adam B Salmon

**Affiliations:** 1 The Geriatric Research Education and Clinical Center, South Texas Veterans Health Care System, Audie L. Murphy Hospital, San Antonio, TX, 78229, USA; 2 Department of Molecular Medicine, The Sam and Ann Barshop Institute for Longevity and Aging Studies, The University of Texas Health Science Center at San Antonio, 15355 Lambda Drive, MSC 7755, San Antonio, TX, 78245-3207, USA

**Keywords:** Oxidative stress, Insulin resistance, Muscle, Adipose, Mitochondria, Inflammation

## Abstract

One of the most common pathologies in aging humans is the development of glucose metabolism dysfunction. The high incidence of metabolic dysfunction, in particular type 2 diabetes mellitus, is a significant health and economic burden on the aging population. However, the mechanisms that regulate this age-related physiological decline, and thus potential preventative treatments, remain elusive. Even after accounting for age-related changes in adiposity, lean mass, blood lipids, etc., aging is an independent factor for reduced glucose tolerance and increased insulin resistance. Oxidative stress has been shown to have significant detrimental impacts on the regulation of glucose homeostasis in vitro and in vivo. Furthermore, oxidative stress has been shown to be modulated by age and diet in several model systems. This review provides an overview of these data and addresses whether increases in oxidative stress with aging may be a primary determinant of age-related metabolic dysfunction.

## Review

### Introduction

The aging process is associated with functional declines in nearly every physiological parameter measured to date [1]. One of the most common physiological declines in human aging is a general deficit in the ability to effectively regulate glucose metabolism. The prevalences of elevated fasting glucose levels, glucose intolerance, and insulin resistance have all been shown to be significantly elevated in the aged population [2-5]. Indeed, one of the most common diseases of the elderly is type 2 diabetes mellitus. The NIH estimated that in 2010 approximately 4% of people aged 20–44 in the United States were affected by type 2 diabetes; in contrast, in this same period 26.9% of people older than 65, or 11 million Americans, were suffering from this disease. Determining how aging so powerfully affects the progression of this disease could lead to potential prevention and reduction in the aged population.

Aging in mammals is generally associated with accumulating adipose tissue, diminishing lean mass and elevated blood lipids, all of which are associated with the development of insulin resistance. Even after controlling for these confounds, aging alone is an independent factor in the declining response to insulin in skeletal muscle and other tissues [6-8]. In a longitudinal study of over 4,500 healthy individuals, Lindstrom et al. showed the likelihood of patients 55–64 years of age for developing drug-treated diabetes was roughly equivalent to patients with a BMI >30 [9]. In other words, clinical obesity and advanced age present nearly the same risk of causing metabolic dysfunction. This is particularly worrisome when considering that several diabetes risk factors (obesity, hyperglycemia, etc.) have straightforward treatment options whereas aging is a persistent, unyielding process. The mechanisms that regulate the effects of aging on glucose metabolism have not yet been clearly identified. Recent evidence has suggested that oxidative stress may be an important factor in this process. The Oxidative Stress Theory of Aging has been one of the most prominent mechanistic theories on how the aging process is regulated. Most studies in this field have shown little effect of oxidative stress on mammalian lifespan, but there is clear evidence that oxidative stress can promote the development of many age-related diseases and pathologies [10].

Oxidative stress is a necessary byproduct of life in aerobic environments. Oxygen free radicals are generated through several cellular processes, the most significant of which occurs with aerobic respiration in the mitochondria. In the presence of molecular oxygen, the reactions that generate ATP in the electron transport chain complexes of the mitochondria can generate oxygen free radicals [11]. However, aerobic organisms have evolved a complex antioxidant defense system to reduce free radicals, prevent oxidative damage, and repair oxidative damage that does occur [10]. An exhaustive review of all cellular pro-oxidant and antioxidant sources are beyond the scope of this review. At the most basic level, oxidative stress occurs under conditions that favor factors that generate oxygen free radicals (pro-oxidants) rather than those factors that evolved to reduce these radicals (antioxidants). In contrast, conditions that favor reduction of free radicals, for example by increasing cellular antioxidants, would be predicted to reduce oxidative stress under normal physiological conditions. The remainder of this review will provide support for the hypothesis that age-dependent alterations in this balance promote oxidative stress which causes metabolic dysfunction.

### Oxidative stress causes metabolic dysfunction

Several studies have shown that oxidative stress can directly promote cellular insulin resistance and alter glucose metabolism. In cell culture, oxidative stress dramatically decreases the cellular response to insulin. In these studies, treatment with oxidative stress significantly reduced insulin-stimulated glucose uptake, reduced phosphorylation of the downstream insulin signaling mediators protein kinase B (AKT), insulin receptor substrate protein-1 (IRS-1), and glycogen synthase kinase-3, and reduced glucose transporter (GLUT4) translocation to the cellular membrane [12-16]. Similarly, glucose transport was reduced in ex vivo rat muscle following oxidative stress [17,18]. Chronic H_2_O_2_ treatment of ex vivo muscle also inhibited insulin-stimulated phosphorylation of the insulin receptor and AKT and caused a selective loss of IRS proteins which further inhibited insulin response [18]. Furthermore, treatment with antioxidants prevented the development of insulin resistance in both cell culture and ex vivo experimental models [15,16,18]. Antioxidants also partially prevented cellular insulin resistance caused by tumor necrosis factor-alpha (TNFα), glucocorticoids, and the saturated fatty acid palmitic acid, suggesting that oxidative stress plays a key role in their action on glucose metabolism [19,20]. Together, these data show that oxidative stress can both directly and indirectly inhibit glucose metabolism.

There is also strong evidence that elevated levels of oxidative stress are correlated with defects in glucose metabolism in both human clinical studies and laboratory models. For example, urinary levels of the DNA oxidation marker 8-oxo-2'-deoxyguanosine (8-OHdG) and plasma levels of the lipid oxidation marker malondialdehyde (MDA) are significantly elevated in diabetic patients [21-23]. One interpretation of this is that hyperglycemia with metabolic dysfunction may promote oxidative stress [24,25]. However, even pre-diabetic patients show elevated urinary levels of 8-OHdG suggesting that oxidative stress may precede the clinical development of glucose intolerance, insulin resistance and diabetes [26]. Similarly, Tinahones *et al.* found the degree of insulin resistance among obese patients was positively correlated with plasma lipid peroxidation levels and inversely correlated with plasma antioxidant levels [27]. Furthermore, defects in glucose metabolism are associated with both reduced levels of antioxidants (glutathione and vitamin E and C) as well as reduced activity of antioxidants (superoxide dismutase, glutathione peroxidase, and glutathione reductase) in plasma [27-32]. Together, these findings hint that oxidative stress may act as both cause and effect of metabolic dysfunction in a vicious cycle towards developing diabetes.

Though there is a clear association between oxidative stress and the pathological state of diabetes (or pre-diabetes), the association between oxidative stress and age-induced metabolic dysfunction is less clear. Samiec *et al.* reported that plasma levels of the antioxidant glutathione from healthy individuals older than 60 years are greater than those of younger diabetics but less than those of healthy younger individuals [30]. Interestingly, this study also reported that levels of the oxidized form of glutathione (GSSG) were relatively high in both healthy older and diabetic individuals [30]. Similarly, Nuttal *et al.* found that plasma lipid hydroperoxide levels were highest in elderly diabetics (~75 years old) followed by healthy elderly (~70 years old) with the lowest levels were reduced in healthy young individuals [33]. This study also showed total antioxidant capacity and glutathione levels were reduced in both elderly diabetics and healthy elderly compared to healthy young [33]. Mendoza-Nunez *et al.* showed that both diabetes and aging are risk factors for increased oxidative stress with an additive effect of both phenotypes [34]. Even among diabetic patients, markers of plasma oxidative stress significantly increase with age [35]. This trend is also evident in laboratory mice; Wu *et al.* found that protein oxidation and activation of oxidative stress-sensitive pathways were elevated in both young diabetic and old mice and elevated further still in old diabetic mice [36]. Examining whether oxidative stress and/or damage is associated with mild age-related metabolic defects, rather than frank diabetes, is an important next step to support the hypothesis of this review.

### Oxidative stress accumulates with age in tissues that regulate glucose metabolism

Glucose metabolism in mammals is regulated by a complex set of endocrine signals that ultimately affect insulin release from the pancreas, glucose production from the liver, and glucose uptake in peripheral tissues including skeletal muscle, adipose, liver, brain, and cardiac muscle. Development of insulin resistance in skeletal muscle, white adipose tissue, and liver is generally thought to be the primary etiology of glucose metabolism dysfunction. The insulin response in each of these tissues shows a significant decline with age [2,3,37]. If oxidative stress is a significant contributor to this process, it would be predicted that elevated levels of oxidative stress/damage in these tissues would be associated with 1) metabolic dysfunction and 2) increasing age.

As discussed above, there is strong evidence supporting the first prediction; that is, circulating levels of oxidative stress are generally elevated in vivo in experimental models of metabolic dysfunction. There is also strong evidence for increased oxidative stress in the tissues of these models. Obesity, whether caused by genetic factors or high fat/high caloric diets, diminishes insulin sensitivity and increases oxidative stress in rodents and humans [38]. For example, skeletal muscle and adipose tissue of high-fat-fed laboratory mice show high levels of protein oxidation as measured by total levels of proteins bound with carbonyl or 4-HNE (4-hydroxenonal) adducts [39,40]. Similarly, lipid oxidation levels are also elevated in these tissues from high fat fed mice and rats [38,41]. Moreover, skeletal muscle mitochondria production of superoxide and hydrogen peroxide is significantly increased with high fat feeding [42,43] as is superoxide production from NADPH oxidase activity in adipose tissue [38]. There is limited data on oxidative stress/damage in tissues from human subjects due to the challenges in obtaining samples. However, obesity in humans is associated with increased protein oxidation adducts in subcutaneous adipose tissue and skeletal muscle [44,45]. Together, these findings show that reduction in glucose metabolism in rodents and humans is strongly associated with oxidative stress.

Since the development of the Oxidative Stress Theory of Aging over 50 years ago, there have been extensive efforts to address whether accumulating oxidative damage (including that in muscle, liver and adipose tissue) is a significant modulator of the aging process [46]. Old mice do have increased levels of DNA, lipid and protein oxidation in skeletal muscle [47-49] and mitochondria from muscles of old mice produce more hydrogen peroxide than those from young mice [47]. Similarly, muscle biopsies from healthy older human subjects show increased levels of lipid peroxidation and reduced levels of several antioxidants [50]. Liver and visceral adipose have also been reported to accumulate lipid and protein oxidative damage with age in both mice and rats [48,51-55].

Oxidative stress might also negatively affect glucose metabolism at the level of insulin release from the β-cells of the pancreas. In comparison to other tissues, the relative expressions of the antioxidants superoxide dismutases (both the cytosolic Cu/Zn SOD and mitochondrial Mn SOD), glutathione peroxidases, and catalase are reduced in islet cells [56,57]. A blunted antioxidant defense may be beneficial in these cells because reactive oxygen species are required for normal β-cell response to glucose [58,59]. However, this phenotype also renders pancreatic β-cells particularly sensitive to the negative effects of oxidative stress. For example, streptozotocin, a toxin selective to β-cells used to create diabetic rodents, works in part by generating oxidative stress [60,61]. Several antioxidant treatments have been shown to prevent streptozotocin-induced β-cell death [60-62]. Furthermore, genetic overexpression of antioxidants has been shown to protect the pancreas in diabetic models [63]. While the effect of aging on oxidative damage in the pancreatic cells has not yet been addressed, there are several reports of increased oxidative damage in this tissue with metabolic dysfunction [60,61,64,65].

**Figure 1:**
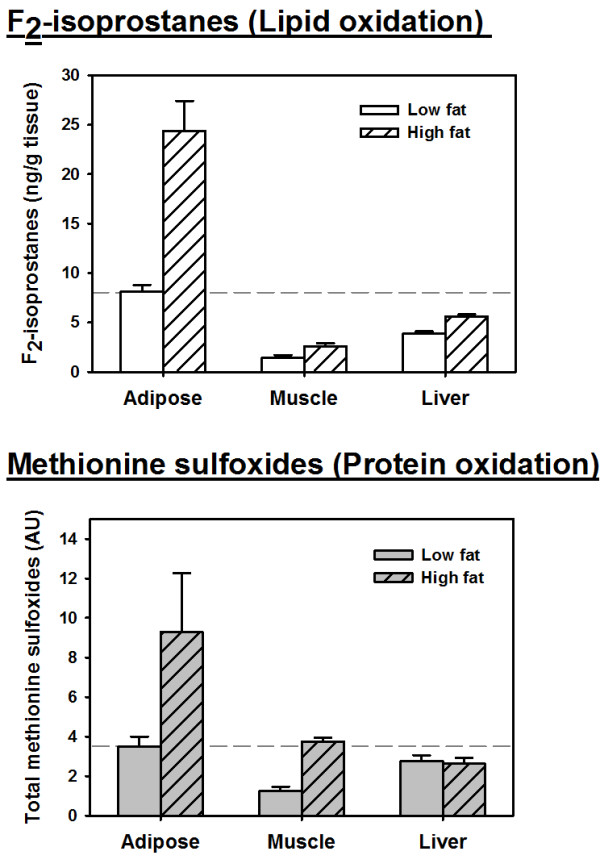
**High oxidative stress in mouse adipose tissue.** Levels of F2-isoprostanes (top) and methionine sulfoxides (bottom) in young male C57BL/6 mice. Bars represent mean levels of indicated oxidation moiety (± SEM) from four animals for each indicated measurement. Samples shown represent epididymal depot/visceral fat (adipose tissue), quadriceps (skeletal muscle), and liver. Dashed line indicates mean value for adipose tissue in low-fat (normal)-fed animals. High-fat diet animals were fed a defined diet with 45% kCal from fat (Research Diets D12451P) for 16 weeks. Isoprostanes were measured as previously described [70]. Methionine sulfoxides were measured by immunoblot using methionine sulfoxide from Oxford Biochemical.

It is likely that all tissues are targets of oxidative damage in metabolic dysfunction and/or aging; however, there is evidence that adipose may be more susceptible to, or generate more, oxidative stress than others. Until recently, white adipose tissue was thought to be a largely inert, energy storing tissue. However, adipose tissue is a dynamic endocrine organ and changes in its function may play a significant role in the aging process in mammals [66]. White adipose tissue, particularly visceral adipose, is characterized as highly inflammatory. Aging significantly increases the production of proinflammatory cytokines from macrophages and pre-adipocytes in this tissue [67-69]. Our own data show that oxidative damage is higher in visceral adipose than either skeletal muscle or liver even at a young age. Levels of lipid peroxidation (measured by F_2_-isoprostanes) in young male C57BL/6 mice were more than two-fold higher in visceral adipose compared to muscle and liver (Figure 1). Levels of protein oxidation (measured by methionine sulfoxides) in adipose tissue were also significantly greater than in muscle or liver (Figure 1). Furthermore, modulation of oxidative stress in this tissue is extremely responsive to environmental conditions. Induction of metabolic stress by high fat feeding dramatically increased both of these markers in adipose tissue with more modest changes in both muscle and liver (Figure 1).

### How does oxidative stress promote metabolic dysfunction?

Oxidative stress may exert its effects on glucose metabolism through three primary pathways: (1) by activation of stress-responsive signaling pathways, (2) by promotion of proinflammation and (3) by directly affecting the insulin signaling pathway. For the purposes of this review, these pathways have largely been studied for their effect on reduction of insulin signaling. For example, activation of the c-jun N terminal kinase (JNK) signaling pathway has been implicated in the etiology of diabetes and insulin resistance, particularly that caused by obesity [71]. Activation of JNK, in particular the JNK1 isoform, phosphorylates IRS-1 at sites that prevent its recruitment to the activated insulin receptor thus preventing propagation of insulin signaling and promoting insulin resistance [72,73]. Oxidative stress can activate JNK signaling both directly and indirectly through several kinase pathways [74-76]. JNK activation is significantly upregulated in obesity and metabolic dysfunction; further, genetic ablation of JNK in mice (either whole body or liver-specific) prevents dietary- and genetically- induced insulin resistance [77,78]. Several tissues have been reported to show elevated JNK activation with aging in both rodents and humans [79,80].

Chronic inflammation has been proposed to significantly influence the progression of several age-related diseases and pathologies, including the decline in glucose metabolism homeostasis [71,81,82]. Oxidative stress promotes the production of several pro-inflammatory cytokines including interleukin-6 (IL-6) and monocyte chemotactic protein in cells [83]. Oxidative stress can also activate nuclear factor κB (NfκB), a transcription factor that regulates production of TNFα and IL-6 [84,85]. Oxidative stress also promotes cellular senescence, particularly in adipocytes; these senescent cells can then promote localized inflammation by secreting proinflammatory cytokines and by recruiting macrophages [86-88]. TNFα and IL-6 have been shown to inhibit insulin signaling through inhibitory phosphorylation of IRS1; ablation of these cytokines prevents insulin resistance in cells and in vivo [89,90]. Aging is a significant factor in the increased expression of multiple proinflammatory cytokines including TNFα and IL-6 [67,91]. Furthermore, aging has been shown to prevent the normal suppression of TNFα that occurs postprandially [92].

It is also possible that oxidative stress may directly cause insulin resistance by damaging cellular components required for proper insulin signaling. Oxidative stress impairs binding of insulin to the insulin receptor and reduces cellular internalization of insulin, presumably by promoting oxidative damage at its binding site [93]. Similarly, Zhou *et al.* and Van den Dobbelsteen *et al.* showed that high doses of peroxynitrite or redox-cycling agents can inhibit the ability of insulin receptor to undergo autophosphorylation [94,95]. Oxidative stress disrupts the insulin-stimulated cellular redistribution of IRS1 and phosphoinositide 3-kinase required for efficient transduction of insulin signaling, GLUT4 translocation and glucose uptake [96]. In addition, GLUT4 levels decrease with oxidative stress because of oxidative damage to nuclear proteins regulating its expression [97]. It has been suggested that age-associated insulin resistance is partly due to diminished function of insulin signaling proteins [98]. Proteins that undergo oxidation have clear and marked declines in function largely due to changes in their structural conformation [99,100]. It still remains to be determined whether the decline in insulin signaling function can be ascribed to this biochemical process.

### Can antioxidants prevent metabolic dysfunction in vivo?

If oxidative stress is a significant cause of glucose intolerance/insulin resistance, antioxidant treatments would be predicted to prevent metabolic dysfunction. In rodent models, there is substantial evidence supporting this hypothesis. For example, treatment of mice with the mitochondria-targeted superoxide dismutase mimetic MnTBAP prevented the development of insulin resistance and glucose intolerance caused by high fat diet feeding [101]. MnTBAP treatment also improved glucose homeostasis in genetically obese mice [19]. Rats treated with a different mitochondria-targeted small molecule antioxidant, SS31, showed a similar protection from high fat diets [42]. Several genetic models of increased antioxidant expression have also been shown to protect from obesity-induced insulin resistance; overexpression of Mn-superoxide dismutase, catalase (specifically in mitochondria; mCAT) and peroxiredoxin 3 have all been shown to preserve glucose homeostasis with high fat feeding [42,101,102]. Antioxidant treatments have also been suggested to prevent age-associated metabolic dysfunction in rodent models. Lee *et al.* showed that mCAT mice are protected from age-associated declines in insulin due to due to reduced mitochondrial H_2_O_2_ production and reduced accumulation of oxidative damage [103]. Long-term treatment of rats with the antioxidants vitamin C or butylated hydroxytoluene improved the insulin response of adipocytes isolated from old animals [104].

Despite the strong evidence that antioxidants may be beneficial for glucose metabolism in rodents, most studies on antioxidant therapy in humans have been largely inconclusive. Classical antioxidant therapies, such as vitamin C and vitamin E, appear to have little effect on metabolic dysfunction of several forms [105]. Other antioxidants such as coenzyme Q10, alpha-lipoic acid, and L-carnitine have also been largely ineffective as treatments [106]. Because these clinical studies only treated individuals exhibiting diabetes or pre-diabetes, one interpretation may be that these treatments cannot *reverse* metabolic dysfunction. If oxidative stress precedes the development of glucose tolerance and insulin resistance [26], it may be more apt to utilize antioxidants as a preventative as in many of the rodent studies above. Furthermore, issues of dosage and bioavailability in target tissues are significant confounds in these studies [107]. For example, because of the high lipid levels in adipose, this highly oxidized tissue may not be strongly affected by water-soluble antioxidants. New insights into the mechanisms by which oxidative stress negatively affects glucose metabolism may provide new antioxidants, or treatment regimes, that can prevent age-related metabolic dysfunction.

## Conclusions

**Figure 2:**
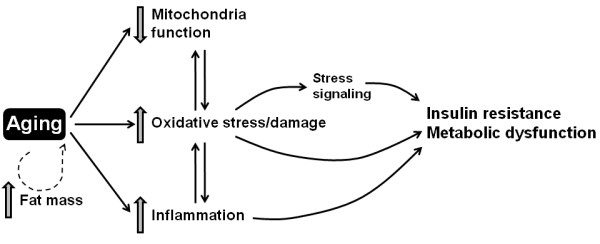
**Proposed model illustrating the potential links between aging, oxidative stress, and metabolic dysfunction** .

The high incidence of metabolic dysfunction in the aged population is a significant health and economic burden on a growing population. The population growth of both individuals over 65 years of age and obese individuals suggests that this problem will not diminish without significant intervention. While improving diet, increasing aerobic exercise and weight loss can all improve glucose metabolism, aging remains an independent factor in the development of insulin resistance [6-8]. Understanding this process could significantly improve both health span of the aging individual and health of the elderly population. How oxidative stress may fit into this process is summarized in Figure 2. Regulating oxidative stress does make for an attractive treatment target; the process is well studied, there are precise assays for its measurement, and there are viable treatments, such as antioxidants, that reduce its effects.

While experimental evidence has suggested that antioxidant treatments can prevent metabolic dysfunction caused by diet or aging in rodents, clinical studies have largely failed. Some potential rationales for these discrepancies have been described above. However, the benefit of healthy diet, activity and lifestyle on reducing oxidative stress in vivo cannot be discounted as a means to at least slow the progression of metabolic defects [108-113]. As mentioned above, studies in rodents suggest that there may be some benefit to increasing antioxidant levels as anti-diabetic prophylactic treatment rather than clinical treatment [19,42,101]. Support would require long-term longitudinal studies to determine whether these treatments would be preventative. In the short-term, new insights into the mechanisms by which oxidative stress negatively affects glucose metabolism may develop new antioxidants, or treatment regimes, that can prevent age-related metabolic dysfunction. For example, new treatments that specifically target muscle or adipose tissue may have much stronger effects on glucose metabolism at lower doses than more general antioxidants. The goal of these studies should not be to find alternatives to healthy living, but rather to utilize treatments in conjunction with healthy lifestyles to extend the health span in humans.

## Abbreviations

8-OHdG: 8-Oxo-2'-deoxyguanosine; AKT: Protein kinase B; GLUT4: Glucose transporter 4; IL-6: Interleukin-6; IRS-1: Insulin receptor substrate protein-1; JNK: c-jun N terminal kinase; MDA: Malondialdehyde; NfκB: Nuclear factor κB; TNFα: Tumor necrosis factor alpha.

## Competing interests

The author declares no financial competing interests.
